# Developing and Testing Remote Implementation for the Changing Talk Online (CHATO) Communication Intervention for Nursing Home Staff: A Pilot Pragmatic Randomized Controlled Trial

**DOI:** 10.1093/geroni/igac026

**Published:** 2022-05-02

**Authors:** Carissa K. Coleman, Maria Hein, Clarissa A Shaw, Tim Beachy, Yelena Perkhounkova, Amy Berkley, Kristine N Williams

**Affiliations:** School of Nursing, University of Kansas Medical Center, Kansas City, Kansas, USA; College of Nursing, University of Iowa, Iowa City, Iowa, USA; College of Nursing, University of Iowa, Iowa City, Iowa, USA; College of Public Health, University of Iowa, Iowa City, Iowa, USA; College of Nursing, University of Iowa, Iowa City, Iowa, USA; School of Nursing, University of Kansas Medical Center, Kansas City, Kansas, USA; School of Nursing, University of Kansas Medical Center, Kansas City, Kansas, USA

**Keywords:** Alzheimer’s disease, Caregiver communication, Elderspeak, Nonpharmacological intervention, Quality improvement

## Abstract

**Background and Objectives:**

The Changing Talk (CHAT) communication training effectively reduces elderspeak and subsequent behavioral challenges in residents with dementia in nursing homes (NHs). As part of the pilot pragmatic clinical trial testing Changing Talk: Online Training (CHATO), a new online version, a remote implementation design, and process evaluation were developed to capture contextual factors, ensure fidelity, and determine effective implementation strategies.

**Research Design and Methods:**

The Expert Recommendation for Implementing Change compilation informed this 2-phase approach to develop and test remote implementation. An Advisory Board guided the developmental phase while pilot testing used a cluster-randomized design. Data were analyzed to evaluate NH characteristics; implementation strategies used; CHATO participation, completion, and passing rates; and leadership evaluation.

**Results:**

Five out of 7 NHs were nonprofit with above average quality ratings (*M* = 4.3 of 5). Staff participants (*N* = 237) were mostly female (90%), non-Hispanic White (91%), and nursing assistants (46%). Implementation time ranged from 54 to 86 days (*M* = 70.3, standard deviation [*SD*] = 9.3), with planning phase ranging from 11 to 29 days (*M* = 20.1, *SD* = 6.7), and training phase ranging from 35 to 58 days (*M* = 50.0, *SD* = 7.6). A range from 3 to 11 implementation strategies were used by each NH. Assigning champions, including the social worker on the implementation team, utilizing multiple mediums for reminders, giving rewards or public recognition, supporting onsite discussions, and other tailoring strategies were associated with improved outcomes. Participation ranged from 20% to 76%. Over 63% of participants completed training (*N* = 150) and 87% passed the posttest (*N* = 130). Leadership evaluations noted staff used CHATO concepts in practice and improved communication culture.

**Discussion and Implications:**

Leadership who took an active role, engaged multiple team members, and varied strategies had better outcomes. Effectiveness of the strategies will be evaluated in a national pragmatic clinical trial testing CHATO’s effects on reducing behavioral and psychological symptoms in dementia care.

**Translational Significance:** This study demonstrates the application of Expert Recommendation for Implementing Change implementation strategies in developing and testing remote implementation for nonpharmacological dementia care interventions. Findings indicate the use of available supports can impact success of interventions and may be critical for optimizing effects of interventions in real-world settings and investment in implementation at the organizational level, engaging multiple team members, and using multiple strategies can improve outcomes when implementing online education. Improving communication through online education has the potential to improve both resident and staff outcomes.

There are an estimated 5.8 million people with Alzheimer’s disease in the United States and 50 million worldwide with a rising projection to 152 million by 2050 (Alzheimer’s Association [AA], 2021; World Health Organization [WHO], 2020). Dementia has significant social and economic implications in terms of direct medical and care costs which are estimated to be roughly $818 billion dollars, equivalent to 1.1% of global gross domestic product (WHO, 2020). Specific to nursing homes (NHs), the cost of dementia care is high due to the intensive level of treatment, poorly coordinated care, staff shortages, and lack of dementia care skills (AA, 2021; Jutkowitz et al., 2017; Mitchell, Black, et al., 2012, Mitchell, Teno, et al., 2009). Care of persons with dementia in NHs is further complicated by behavioral and psychological symptoms of dementia (BPSD), such as aggression, vocal outbursts, wandering, and withdrawal that occur as persons with dementia lose the cognitive and communication abilities to express their unmet physical and psychosocial needs (Kales et al., 2015; Kovach et al., 2005).

Resistiveness to care (RTC) is a type of BPSD that increases staff stress and time to complete care, often leading to staff turnover, injury, and inappropriate use of psychotropic medications to control symptoms. As NH residents become unable to convey care preferences and needs, staff communication becomes infantilizing, impersonal, and task-oriented resulting in BPSD (Williams, 2006; Williams et al., 2005). NH residents are twice as likely to exhibit RTC when staff use elderspeak, a communication that sounds like baby talk, compared to normal communication (Williams et al., 2009). Thus, improving communication has great potential as an inexpensive nonpharmacological intervention to reduce BPSD in NH care (Eggenberger et al., 2013; Kales et al., 2019). The Changing Talk: Online (CHATO) training educates NH staff to use appropriate methods of communication resulting in less elderspeak, more person-centered communication, and subsequent reduction in BPSD and psychotropic medication use.

## Background

The in-person Changing Talk (CHAT) communication training was originally developed to educate NH staff about elderspeak’s negative effects and guide practice to more effective, person-centered communication. It consisted of three, 1-hr long, in-person classroom sessions over 3 weeks. CHAT has confirmed effects on communication in three studies among staff and residents in over 20 NHs. Based on behavioral coding of video recordings, both elderspeak and RTC declined at postintervention and after 3-month follow-up (Williams et al., 2017). Linear mixed modeling determined change in elderspeak was significantly predicted by CHAT and baseline elderspeak, while RTC change was significantly predicted by elderspeak change, baseline RTC, and resident comorbidities. The average proportion of elderspeak in staff-resident interactions have ranged from 35% to 58% with some staff using elderspeak in 99% of their interactions (Herman & Williams, 2009; Williams, 2006; Williams et al., 2003, 2017). Effect sizes for CHAT ranged from η ^2^ = 0.35 to 0.62 for reducing diminutives (inappropriately intimate terms of endearment) and collective “we” pronoun substitutions. Person-centered communication instead of task-focused topics has also increased with CHAT (Williams et al., 2005).

Despite the success of CHAT in reducing elderspeak and RTC, common education barriers exist related to staff turnover, absenteeism, heavy workloads, and personal conflicts (Banazak et al., 2000; Beeber et al., 2010; Low et al., 2015; Williams et al., 2016, 2017). Although CHAT sessions were held on multiple days and times, staff found it difficult to attend all three classes (Williams et al., 2017). Because the classroom format limited staff access and feasibility for widespread dissemination, an online web conference version of the training with multiple NHs was offered; however, engagement of individual staff was limited with this approach (Coleman et al., 2015). To facilitate dissemination, the CHATO training was developed to provide all the CHAT training content with asynchronous and independent access for busy staff (Williams et al., 2017). An instructional designer, item writer, and media team were assembled to transition CHAT content, including 20 video clips of NH staff-resident interactions, to the online CHATO modules (Williams et al., 2017). Scripts from the original CHAT were narrated to maintain the content, eliminating a need for advanced literacy skills. Interactive scenarios and game-based activities were added to engage staff as well as a virtual discussion forum after each module.

The newly developed CHATO modules were tested for usability, and a new 13-question knowledge gain test, the Changing Talk Scale (CHATS) was also developed and tested in the pilot pragmatic clinical trial. To pilot test CHATO in NHs without an onsite interventionist, remote implementation and a process evaluation were necessary to capture environmental factors and their impact on training outcomes. Implementation included the need to engage NH leadership to motivate and inspire targeted staff, to be easy and accessible; and ensure consistent application across NHs while also being adaptable to the individual NH’s needs and preferences. The process evaluation had to be remotely driven while capturing contextual factors and strategies chosen by each NH. Once developed, remote implementation and the process evaluation were pilot tested to prepare for a national pragmatic clinical trial to test CHATO’s primary resident outcomes, BPSD and psychotropic medication reduction for persons with dementia, and the impact of implementation on these outcomes. The purpose of this report is to describe the CHATO pilot implementation development and process and determine the facilitators, barriers, and outcomes related to our remote implementation.

## Method

Planning for the remote implementation went through two phases informed by the Expert Recommendation for Implementing Change (ERIC) strategy compilation (Glasgow et al., 2019; Powell et al., 2015). The first phase focused on the development of the supports and materials with Advisory Board oversight. The second phase refined implementation during pilot testing of CHATO using a cluster-randomized design with immediate and wait-list control groups. Ethical approval was received from the University of Kansas IRB (STUDY00142916).

### CHATO Intervention

The new online training is approximately 3 hr long, divided into three modules. The modules are narrated by a PhD-prepared nurse educator and professor. Individual staff log into an online learning management system and move through each module, each module building on the content of the previous module. Module 1 contains information on the importance, benefits, and components of effective communication. Module 2 focuses on common communication barriers and challenges, elderspeak communication, and effective and ineffective communication strategies. Module 3 addresses common problems during communication, guidelines for improving communication, and characteristics of person-centered communication. The training contains interactive learning activities, a virtual discussion forum after each module to share experiences and reflect with others, pre and posttests, and evaluations. Practice activities provided in each module provide opportunities to apply the knowledge and skills while interacting with residents during daily routines. The goal of CHATO is to increase awareness of the importance of effective communication with older adults and to use evidence-based, person-centered communication during interactions with older adults in NHs and other residential care settings. The CHATO training is designed for staff in NHs and health care settings in the community that include registered nurses, nursing assistants, dieticians, direct-care professionals, and other administrative and support employees.

## Development

### Implementation Design

Quarterly meetings were conducted with an Advisory Board and NH industry consultants from September 2018 to August 2019 to gather input and feedback on implementation and the support materials. The board consisted of NH administrators, directors of nursing, nurse training specialists, and a marketing consultant. Additional consultant input was provided by leading age, a nationally recognized consortium of aging advocates, educators, and researchers. Field notes were taken at meetings and used to modify materials. Ongoing quarterly advisory committee meeting topics are listed in Table 1.

**Table 1.** Advisory Board Topics and Discussions for Developing Remote Implementation

Meeting	Topics
1	Recruitment and marketing ideas; implementation strategies and tailoring to individual nursing homes
2	Implementation timeline including number of meetings with leadership (at least three with weekly technical assistance); mandatory training with incentive suggested; marketing and communication plan, recruitment ideas, and surveys reviewed
3	Reinforcing and maintaining staff skills over time; ideas included tools to embed in orientation, booster sessions, onsite discussions, pocket guides; online training is preferred due to ease of access and staff autonomy
4	Behavior and medication outcome data, process evaluation, fidelity checklist, possible data collection for cost, adoption, maintenance, and sustainability as they related to the national pragmatic trial

A 90-day implementation timeline was determined to be optimal including three 1-month phases: planning, training, and follow-up. At the initial meeting for each NH, an Implementation Lead was identified, typically the director of nursing (DON). During the planning phase, strategies would be tailored to the organizational culture and current practices. During the training phase, weekly participation and completion rates were shared with the Implementation Lead. After the training closed, a closeout meeting with NH leadership was held to gather feedback. Surveys and interviews were scheduled during the follow-up phase (Table 2). Technical assistance was provided through all phases.

**Table 2.** Research Implementation Overview and Timeline

Phase	Timeline	Participants	Overview
Development	September 2018–August 2019	Four advisory board meetings Four consultant meetings	Developed staff engagement methods Developed implementation materials •Website •Communication plan •Implementation toolkit Developed training manual Developed fidelity checklist Developed evaluation
Feasibility	September 2019–October 2019	One NH (NH0)	Initial testing of •Training platform •Remote implementation plan •Fidelity checklist •Evaluation
Pilot testing			
Implementation phases (per NH)	December 2019–April 2020	Eight NHs (NH1–NH7) (1 unable to start due to COVID-19 pandemic)	
Planning			Initial virtual meeting topics •Identify eligible staff •Assign Implementation Lead •Overview of training Provided implementation materials Completed fidelity checklist
Training			Provided weekly participation reports Provided technical assistance Scheduled virtual meetings as needed Completed fidelity checklist
Follow-up			Closeout virtual meeting topics •Feedback •Evaluation overview Completed fidelity checklist Sent NH surveys •Artifacts of culture change •Implementation strategies Completed evaluation •NH leadership evaluation survey •NH leadership phone interviews (unable to conduct interviews due to COVID-19 pandemic)

*Note:* COVID-19 = coronavirus disease 2019; NH = nursing home; NH0 = feasibility nursing home.

### Implementation Supports

Multiple supports for the NH to use while implementing the training were designed. These included a website, communication plan, implementation toolkit, and training manual. The CHATO website was the main information source for the NHs (Changing Talk: Online (CHATO) Website, 2020). It provided an overview of the training and access to all materials. The implementation toolkit provided possible strategies NHs could use during all phases of implementation (Table 3). For the planning phase, this included identifying champions, informing stakeholders, and planning logistics; for the training phase, discussion formats, modeling and coaching, and staff engagement tools for reminding and rewarding staff; and for the follow-up phase, methods for maintenance and sustainability. The communication plan provided tips for informing stakeholders and staff, posters for advertising, and example text for staff reminders across mediums (e.g., text, email, and social media). Finally, the training manual summarized the key elements of the training to assist staff in leading both virtual and/or onsite discussions.

**Table 3.** Implementation Toolkit Strategies With Corresponding ERIC Strategies

Phase	CHATO strategies	Description	ERIC strategies
Planning	Startup checklist	The startup checklist provided NH leadership with a quick reference of how to implement the training and the core activities in each phase to use throughout the implementation process.	Develop a formal implementation blueprint; assess for readiness and identify barriers and facilitators; develop and implement tools for quality monitoring
	Aligning goals	All staff were encouraged to take the training to increase the likelihood of changing the communication culture of the NH. Leadership was given early access to take CHATO and were also encouraged to link the concepts to other organizational values or QI initiatives to ground the training in larger organizational goals.	Capture and share local knowledge; recruit, designate, and train for leadership; develop and organize quality monitoring systems; purposely reexamine the implementation
	Communication plan	The communication plan involved engaging multiple groups throughout the NH. NHs were advised to inform stakeholder groups across mediums (i.e., social media, presentations, newsletters, etc.) to gain support from residents and their families and create buy-in for staff. Posters were available to download from the CHATO website for adverting and reminding efforts. Example text for reminders were also included.	Build a coalition; conduct educational meetings; involve patients/consumers and family members; remind clinicians
	Champions	Multiple staff were asked to participate in implementation including naming an Implementation Lead and champions. The champions would lead in modeling training concepts in practice, utilizing education supports, informal discussions, and encouraging completion. Leaders were also asked to seek staff input on current barriers impacting completion. Utilize CHATO research team for technical assistance.	Identify and prepare champions; create new clinical teams, develop educational materials; organize clinician implementation team meetings; develop academic partnerships; centralize technical assistance; use an implementation advisor
	Capacity and logistics	Internet and computer access were necessary for completion. Leaders were asked to strategize when and where staff would complete the training (while at work or at home) or how staffing might be impacted. Review and tailor implementation to their nursing home.	Tailor strategies; promote adaptability
Training	Staff engagement	Engagement strategies such as contests, rewards, public recognition as well as adequate advertisement, and regular weekly reminders were encouraged. Advertise contact hours or require certificate for file.	Alter incentive/allowance structures; change accreditation or membership requirements
	Modeling and coaching	Champions assisting with implementation would model effective communication and other CHATO concepts while direct-care supervisors would provide supportive reinforcement and feedback (rather than punitive) to individual staff.	Model and simulate change; provide clinical supervision; identify early adopters; intervene to enhance uptake and adherence
	Discussions	Four types of discussions were highlighted: CHATO virtual discussion board; one-on-one coworker discussions, staff meeting mini-discussions, and onsite group discussions or learning circles.	Create a learning collaborative; facilitation; make training dynamic
Follow-up	Maintain	Ongoing recognition for staff who use CHATO concepts, staff led booster sessions, ongoing staff, family, or resident reflection, or discussions.	Conduct ongoing training; obtain and use patients/consumers and family feedback; use train-the-trainer strategies
	Sustain	Suggested embedding concepts in policies, procedures, and staff evaluation. Share with colleagues.	Inform local opinion leaders; involve executive boards; obtain formal commitments; provide ongoing consultation

*Note:* CHATO = Changing Talk: Online Training; ERIC = Expert Recommendation for Implementing Change; NH = nursing home.

### Implementation Fidelity and Evaluation

A mixed-method evaluation was designed to include surveys completed by the Implementation Lead and the NH administrator to capture NH characteristics, organizational factors, implementation strategies use, and process evaluation. Leadership phone interviews were designed with Leading Age acting as an external evaluator to capture qualitative aspects of each NH’s unique experiences. Due to the onset of the COVID-19 pandemic, these interviews were not completed. Implementation fidelity was ensured by a checklist completed by the research team to standardize and document interactions with each NH. The checklist was completed during each phase of implementation and field notes documented any meetings with NHs (Supplementary Material).

## Pilot Testing

### Setting and Participants

One NH was recruited for feasibility testing, and eight NHs were recruited for the pilot. The NHs were selected from a list of NHs expressing interest in CHATO participation that had been previously recruited by direct contact or through professional organizations. NHs were selected if they had at least 30 eligible staff and no prior CHAT training. A signed Letter of Agreement was obtained from all participating NHs indicating their willingness to participate in the study by implementing the training. Staff were eligible to participate in the study if they were permanent employees and over the age of 18. Staff indicated their willingness to participate in the study by reading and agreeing to a consent statement prior to beginning the training modules.

### CHATO Pilot Trial Design

**Figure 1.**
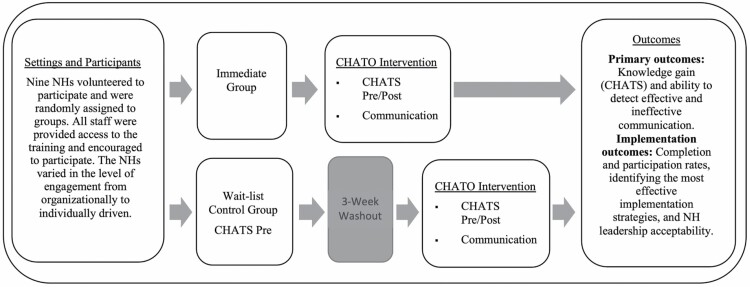
Illustration of the CHATO trial overview. CHATO = Changing Talk: Online Training; CHATS = Changing Talk Scale; NH = nursing home.

CHATO was pilot tested between September 2019 and April 2020 using a cluster-randomized design. NHs were matched based on size. A coin flip within each pair determined group assignment. The immediate group completed the training first. The wait-list control group crossed over to the intervention after a 3-week washout period. A trial overview can be seen in Figure 1. Additional design information and primary outcome results can be found in Williams et al. (2021).

### Implementation Outcomes

The implementation outcomes and process evaluation data were collected from the training modules and through web-based surveys. Phone interviews after the training phase were planned but not completed due to the administrators being too busy with the beginning of the COVID-19 pandemic in March 2020. The measures included the Artifacts of Culture Change Tool, participation and completion rates, an implementation strategies survey, and a leadership evaluation survey.

NH environment and organizational practice were measured using the Artifacts of Culture Change Tool, a 79-item assessment with six subscales: Care Practice, Environment, Family and Community, Leadership, Workplace Practice, and Staffing Outcomes and Occupancy. Responses for each item range from 0 to 5 depending on the scoring for each question and summed for each subscale; the total score is calculated as a sum of the subscales (Schoeneman & Bowman, 2006). The Artifacts of Culture Change was created by a CMS collaboration with the Pioneer Network to create benchmarks for administrative, procedural, and structural changes NHs make to create a more home-like, environment for NH residents. Due to the length of this scale, the REDCap survey provided each NH with their subscale scores and total score in comparison to national samples to encourage completion.

NH participation and completion rates were collected in the training platform. Participation rate was calculated as the percentage of enrolled participants of all eligible participants. The completion rate was calculated as the percentage of enrolled participants completing the CHATS posttraining. The implementation strategies survey is a 35-question descriptive survey developed by the investigators with Advisory Board input, to identify the strategies and approach types used by the NH to implement the training. The leadership evaluation survey consisted of nine questions answered by the NH administrators and the CHATO Implementation Lead. Eight items were for the NH-level CHATO evaluation, and one question assessed motivation to participate in the research.

**Figure 2.**
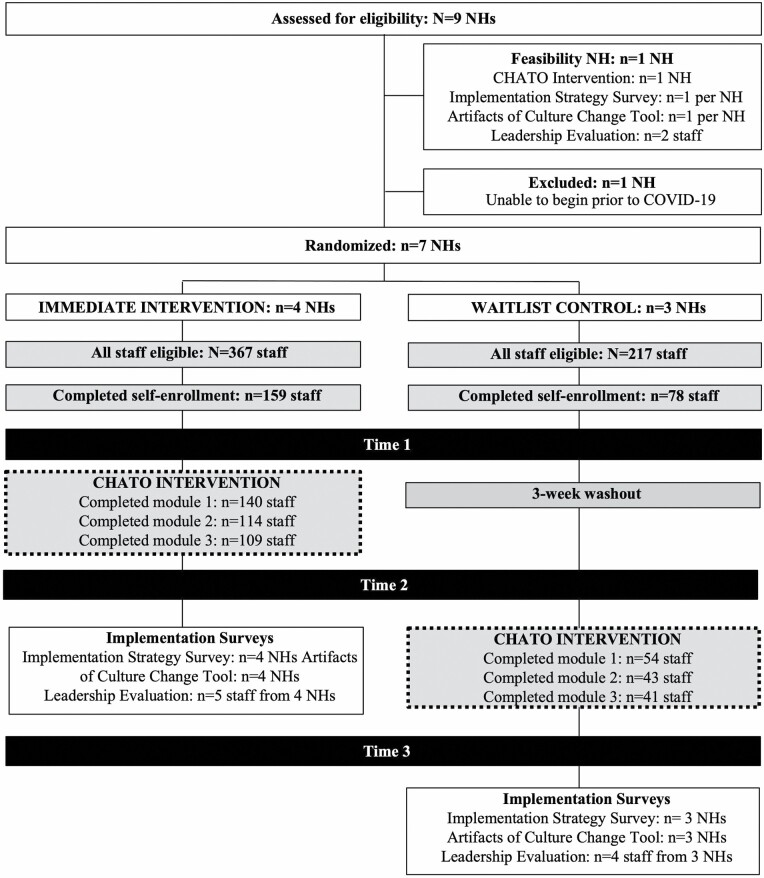
Consort diagram. CHATO = Changing Talk: Online Training; NH = nursing home.

The Artifacts of Culture Change Tool, implementation strategies survey, and leadership evaluation surveys were administered in REDCap (Harris, Taylor, Minor, et al., 2019; Harris, Taylor, Thielke, et al., 2009). The leadership interviews were semistructured, 1-hr interview protocols designed by the investigators and LeadingAge evaluators to capture overall perception, perceived impact, and sustainability of training concepts. The LeadingAge evaluators planned to conduct the interviews via phone 1 month following completion of the training but were not completed due to the start of the COVID-19 pandemic crisis. The consort diagram for the NH-level implementation outcomes is presented in Figure 2.

### Analysis

Demographic and training data were downloaded from the training platform into Excel, and survey data were downloaded from REDCap into Excel. Both data sets were imported and analyzed in SAS Version 8.4 (SAS Institute Inc.). Descriptive analysis was performed using means and standard deviations (*SD*) or frequencies and percentages as appropriate. Participants who completed the training were compared to participants who did not complete the training with respect to their demographic characteristics using Fisher’s exact test.

## Results

Between September 2019 and April 2020, the feasibility NH (NH0) and the seven pilot NHs (NH1–NH7) participated in pilot testing approved by the University IRB. The feasibility NH was used to test the training, the training platform, and remote implementation. Minor modifications were made to the training platform, data collection methods, and implementation to improve performance and/or participation prior to the pilot trial. Pilot testing began in December 2019. One NH was unable to participate due to the start of the COVID-19 pandemic and a lack of staff to dedicate to implementation leadership.

The fidelity checklist was completed by the research team for all NHs during each phase to ensure all received the same information and instruction; however, NHs varied by implementation strategies chosen. All staff at the NH were eligible and encouraged to take the training and the analysis is based on all staff who participated.

### Participants

#### Feasibility NH and staff

The feasibility NH was a small, 5-star nonprofit NH from the Midwest with a staff turnover rate of 26%. The NH averaged 30 occupied beds and cared for mostly non-Hispanic White residents (96.7%) with a third having a dementia diagnosis (31%). The NH did not have a special care unit. Staff participants were typically certified nursing assistants (CNAs, 64.0%) or registered nurses (RNs, 16.0%). They were mostly non-Hispanic White (88.0%) females (92.0%), 41.3 years (*SD* = 13.3) of age on average. The care staff experience averaged 5.6 years (*SD* = 5.4) in their current roles and 9.6 years (*SD* = 9.5) in the current NH. The Implementation Lead was the DON. She is non-Hispanic White, 34 years old, with 4 years’ experience in her role and 8 years at the current NH. The NH administrator is male, non-Hispanic White, 35 years old, with 13 years’ experience in his role and 3 years at the current NH.

#### Pilot NHs and staff

The seven pilot NHs were from six states in the Midwest and West regions of the United States, and five of them were nonprofit. The NHs were rated as above average with the NH Compare quality indicator averaging 4.3 stars and had an average staff turnover rate of 29% (*SD* = 11.6, range = 6.6%–52.0%) for those five that reported it. The NHs averaged 65 beds (range = 30–117 beds) and cared for mostly non-Hispanic White residents (91.1%) with an average of 44.6% (*SD* = 21.3, range = 29%–92%) with a dementia diagnosis. Five NHs did not have a special care unit. Demographic characteristics of staff who enrolled in the study are reported in Table 4. Staff participants enrolled in CHATO were CNAs or certified medical assistants (CMAs, 45.6%), followed by RNs (22.8%) and licensed practical nurses (LPNs, 7.2%). The additional 24.4% of the staff ranged in roles from administrative to direct care. Participants were mostly non-Hispanic White (80.6%), females (89.5%), and 43.2 years (*SD* = 13.6) of age on average. The staff experience averaged 3.8 years (*SD* = 1.6) in their current role and 3.3 years (*SD* = 5.8) at their current NH. There were no significant differences between those staff who completed the training and those who did not complete the training with respect to demographic characteristics, except for ethnicity (*p* = .03). The majority (67.4%) of non-Hispanic or Latino participants completed the training while less (48.8%) than half of Hispanic or Latino participants completed the training.

**Table 4.** Demographic Characteristics of Self-Enrolled CHATO Pilot Participants

Variable	All	Completers	Noncompleters	*p* ^a^
	*N* = 237	*n* = 150	*n* = 87	
	*n* (%)	*n* (%)	*n* (%)	
Age^b^				.44
<25 years	23 (9.7)	11 (47.8)	12 (52.2)	
25–40 years	79 (33.3)	50 (63.3)	29 (36.7)	
41–54 years	78 (32.9)	52 (66.7)	26 (33.3)	
55 years or older	56 (23.6)	36 (64.3)	20 (35.7)	
Missing	1 (0.4)	1 (100.0)	0 (0.0)	
Gender^c^				.37
Female	212 (89.5)	137 (64.6)	75 (35.4)	
Male	24 (10.1)	13 (54.2)	11 (45.8)	
Prefer not to answer	1 (0.4)	0 (0.0)	1 (100.0)	
Race^d^				.09
White	191 (80.6)	126 (66.0)	65 (34.0)	
Asian	7 (3.0)	4 (57.1)	3 (42.9)	
American Indian/Alaska Native	4 (1.7)	2 (50.0)	2 (50.0)	
Black or African American	3 (1.3)	1 (33.3)	2 (66.7)	
Native Hawaiian or other	1 (0.4)	1 (100.0)	0 (0.0)	
More than one race	10 (4.2)	5 (50.0)	5 (50.0)	
Unknown/not reported	21 (8.9)	11 (52.4)	10 (47.6)	
Ethnicity^e^				.03
Non-Hispanic or Latino	172 (72.6)	116 (67.4)	56 (32.6)	
Hispanic or Latino	41 (17.3)	20 (48.8)	21 (51.2)	
Unknown/not reported	24 (10.1)	14 (58.3)	10 (41.7)	
Role^f^				.06
CNA/CMA	108 (45.6)	60 (55.6)	48 (44.4)	
RN	54 (22.8)	42 (77.8)	12 (22.2)	
LPN	17 (7.2)	9 (52.9)	8 (47.1)	
Administration	14 (5.9)	9 (64.3)	5 (35.7)	
Housekeeping	10 (4.2)	9 (90.0)	1 (10.0)	
Dietary	7 (3.0)	2 (28.6)	5 (71.4)	
Social worker	5 (2.1)	5 (100.0)	0 (0.0)	
Activities	4 (1.7)	3 (75.0)	1 (25.0)	
Therapy (PT/OT/speech)	3 (1.3)	2 (66.7)	1 (33.3)	
Support staff	3 (1.3)	2 (66.7)	1 (33.3)	
Educator	1 (0.4)	1 (100.0)	0 (0.0)	
Other	11 (4.6)	6 (54.6)	5 (45.4)	
Highest education^c^				.15
High school or less	58 (24.5)	32 (55.2)	26 (44.8)	
Associate degree or some college	134 (56.5)	85 (63.4)	49 (36.6)	
Bachelor’s degree or higher	36 (15.2)	27 (75.0)	9 (25.0)	
Prefer not to answer	9 (3.8)	6 (66.7)	3 (33.3)	
Years in role^b^				.82
Less than 5 years	95 (40.1)	60 (63.2)	35 (36.8)	
5 to <10 years	48 (20.3)	32 (66.7)	16 (33.3)	
10 years or older	85 (35.9)	52 (61.2)	33 (38.8)	
Missing	9 (3.8)	6 (66.7)	3 (33.3)	

*Notes:* CHATO = Changing Talk: Online Training; CMA = certified medical assistant; CNA = certified nursing assistant; LPN = licensed practical nurse; OT = occupational therapy; PT = physical therapy; RN = registered nurse. Percentages may not sum to 100.0% due to rounding.

^a^ *p* = *p* value for the Fisher’s exact test.

^b^Missing were not included in calculation of the *p* value.

^c^“Prefer not to answer” were not included in calculation of the *p* value.

^d^White participants were compared to all other categories combined.

^e^Unknown/not reported were not included in calculation of *p* value.

^f^CNA/CMA, LPN, RN, and administration were combined into one category and compared to all other categories combined (housekeeping, dietary, social worker, activities, therapy, support staff, educator, and other).

The NHs were encouraged to recruit and enroll all staff in the NH to improve communication throughout the organization. Although, direct-care nursing staff were primarily targeted for the training, additional staff in roles such as housekeeping, social worker, activities, therapy, and support staff also participated. These staffs are not normally provided with continuing education focused on direct care and demonstrated higher completion rates, suggesting their interest in dementia skill development opportunities. The relatively lower completion rates for direct-care staff were likely due to time restraints and competing demands and are not unusual for nonmandatory training (Table 4).

The NH roles of the Implementation Leads are CNA (*n* = 1), RN (*n* = 1), DON (*n* = 3), and administrator (*n* = 2). Most are female (*n* = 5), all are non-Hispanic White, 37.2 years (*SD* = 10.1) of age on average, with a mean of 6.0 years (*SD* = 5.9) in their current role and 3.8 years (*SD* = 2.1) at the current NH. Of the administrators, three are male (*n* = 3), and all are non-Hispanic White, 58.4 years (*SD* = 8.9) of age on average, with a mean of 24.3 years (*SD* = 17.4) in their current roles and 10.2 years (*SD* = 6.8) at the current NH. Two NHs chose not to involve the administrator in the pilot study.

### Primary Outcomes of CHATO Trial

Primary outcomes for pilot CHATO testing included knowledge scores and communication ratings of a video-recorded interaction compared between pre and posttraining. Primary outcomes showed significant improvement posttraining, while participant evaluation was comparable to the original CHAT training. Knowledge increased from a mean pretest score of 61.9% (*SD* = 20.0) to a mean posttest score of 84.6% (*SD* = 13.5) for all participants in the immediate and wait-list control crossover (*N* = 130). Knowledge also significantly improved for the immediate intervention participants (*N* = 95) compared to the wait-list control participants (*N* = 64), pretest to posttest. Ability to recognize ineffective communication improved significantly after training for both groups. A full description of the primary outcome results can be found in Williams et al. (2021).

### Implementation Outcomes

#### NH environment and organizational practices

The Artifacts of Culture Change Tool was used to measure the culture change effort of participating NHs and provided contextual information into training performance. National means for total scale score and subscales were provided by the tool developer, The Pioneer Network, through personal correspondence. The total score ranged 35%–80% across the pilot NHs, compared to a national mean of 59.3% (Table 5). The pilot NHs scored similarly to the national means on the Care Practice and Environment subscales, at 73.6% and 49.1%, respectively, indicating similar levels of resident choice and personalized environments. The mean score of 73.7% on the Family and Community subscale demonstrated a somewhat greater effort than NHs nationwide (66.7%) to include Family and Community in resident life, including intergenerational programs and home-like settings for visiting family. The mean Leadership (47.2%), Workplace Practice (53.6%), and Staffing (69.1%) subscale scores were lower than the corresponding national means, suggesting a more traditional, hierarchal NH with less family, resident, and staff input in decision-making, less flexibility for staff, and higher staff turnover/less staff consistency for residents.

**Table 5.** Measure of Nursing Home Environment: Artifacts of Culture Change

Subscale	National mean	Sample mean	NH0	NH1	NH2^a^	NH3	NH4^a^	NH5	NH6^a^	NH7
Care Practice	74.3%	73.6%	80.0%	74.3%	87.1%	68.6%	90.0%	41.4%	65.7%	81.4%
Environment	48.1%	49.1%	26.3%	59.1%	60.3%	89.4%	79.7%	25.3%	18.1%	34.4%
Family and Community	66.7%	73.7%	60.0%	83.3%	100.0%	66.7%	76.7%	83.3%	60.0%	60.0%
Leadership	56.0%	47.2%	40.0%	60.0%	72.0%	20.0%	72.0%	0.0%	60.0%	52.0%
Workplace Practice	62.9%	53.6%	50.0%	61.4%	70.0%	47.1%	71.4%	44.3%	32.9%	51.4%
Staffing Outcomes and Occupancy	87.7%	69.1%	92.3%	78.5%	56.9%	23.1%	89.2%	66.2%	67.7%	78.5%
Artifacts of Culture Change total scale score	59.3%	56.0%	45.3%	64.7%	66.9%	70.2%	80.5%	36.0%	35.2%	49.1%

*Notes:* Artifacts of Culture Change scale consists of six subscales composed of multiple-choice questions: Care Practice Subscale (14 questions), total = 70; environment subscale (27 questions), total = 320; family and community subscale (six questions), total = 30; leadership subscale (five questions), total = 25; workplace practice subscale (14 questions), total = 70; staffing outcomes and occupancy subscale (13 questions), total = 65. Artifacts of culture change total score is calculated as the sum of subscale scores. The national means were provided by the developer, *The Pioneer Network*.

^a^Wait-list control NHs.

#### NH participation

The CHATO training was offered to all staff at each of the NHs. The participation rate for all staff ranged from 19.5% to 75.7% across all pilot NHs, averaging 40.6%. Once the participants enrolled, the mean completion rate was 63.3% with most participants completing Module 1 (81.9%) and fewer completing Module 2 (66.8%) and Module 3 (63.3%). The time spent in modules was similar for Modules 1 and 2 (mean 78.1 and 70.9 min, respectively), while Module 3 took less time at mean 51.7 min. This aligns with the module content in that Modules 1 and 2 are information and activity-based, while Module 3 is application-based. Roughly one third of participants completed the discussion board in each module. While it was a requirement to enter the virtual discussion to move to the next Module, it was not a requirement to post answers to questions or discuss content with other participants. For the participants completing the training, the passing rate (scoring a minimum of 70% on the posttest) ranged from 71.4% to 100% across the pilot NHs, with a mean of 86.7% (Table 6).

**Table 6.** Nursing Home Participation

Enrollment, completion, and participation rates	All staff	Immediate intervention (*n* = 4)	Wait-list control (*n* = 3)
Eligible participants: *N*	584	367	217
Enrolled participants: *n*	237	159	78
Participation rate: %	40.6	43.3	35.9
Completion rate: *n* (%)	150 (63.3)	109 (68.6)	41 (52.6)
Passing rate: *n* (%)	130 (86.7)	93 (85.3)	37 (90.2)
Module 1			
Completion rate: *n* (%)	194 (81.9)	140 (88.1)	54 (69.2)
Discussion board participation: *n* (%)	76 (32.1)	52 (32.7)	24 (30.8)
Time in module: mean minutes (*SD*)	78.1 (113.0)	80.3 (128.9)	72.1 (48.4)
Module 2			
Completion rate: *n* (%)	157 (66.2)	114 (71.7)	43 (55.1)
Discussion board participation: *n* (%)	75 (31.7)	53 (33.3)	22 (28.2)
Time in module: mean minutes (*SD*)	70.9 (75.4)	66.2 (62.2)	83.6 (102.8)
Module 3			
Completion rate: *n* (%)	150 (63.3)	109 (68.6)	41 (52.6)
Discussion board participation: *n* (%)	71 (30.0)	51 (32.1)	20 (25.6)
Time in module: mean minutes (*SD*)	51.7 (57.9)	46.2 (48.9)	65.2 (74.5)

*Notes:* Enrollment is based on consent and completion of a demographic questionnaire. Participation rate is the percentage of enrolled participants from eligible participants. Completion rate is the percentage of enrolled participants completing the posttest. Passing rate is the percentage of completers (enrolled participants completing the posttest) who scored 70% or better.

#### NH implementation strategies

Across the seven pilot NHs, the total implementation time ranged from 54 to 86 days (mean = 70.3, *SD* = 9.3), with planning phase ranging from 11 to 29 days (mean = 20.1, *SD* = 6.7) and training phase ranging from 35 to 58 days (mean = 50.0, *SD* = 7.6). The implementation strategies varied across NHs (Table 7). Implementation strategies observed in NHs with significant improvements in knowledge gains were: (a) assigning champions (*N* = 1), (b) including the social worker on the implementation team (*N* = 1), (c) utilizing all four mediums (signs, text, email, and verbal) for weekly reminders (*N* = 1), (d) giving rewards or public recognition (*N* = 3), (e) supporting onsite discussions (*N* = 3), and (f) tailoring strategies to their specific NH (*N* = 2). NHs that did not show significant changes in primary outcomes reported a more hands-off approach. They had less experienced Implementation Leads and did not engage with staff at an organizational level (i.e., no onsite discussions, rewards, recognition, or little accountability). Leadership who took ownership of the training, engaged multiple team members, and varied their implementation strategies had better outcomes overall.

**Table 7.** Implementation Strategies Selected by Nursing Homes

Implementation	NH0^a^	NH1	NH2^a^^,^^b^	NH3	NH4^a^^,^^b^	NH5^a^^,^^b^	NH6^a^^,^^b^	NH7^a^^,^^b^
Total time: days	54	65	77	71	72	71	86	66
Planning phase: days	19	11	29	17	25	13	28	19
Training phase: days	35	54	48	54	47	58	58	47
Implementation Lead	DON	CNA	Admin	DON	Admin	DON	RN	DON
Strategies	Leadership takes CHATO. Team (4). All staff. CHATO posters. Self-paced. Lead virtual discussion. **Reward―Gift certificate.**	Leadership takes CHATO. Team (6). All staff. CHATO posters. One module per week. Policy changes. Planning booster sessions.	Leadership takes CHATO. Team (4). All staff. **Champions.** CHATO posters. **Reminded weekly (signs, text, email, verbal).** One module per week. **Reward―Party.** Added to orientation materials. Policy changes. Planning booster sessions.	Self-paced. CHATO posters. Lead virtual discussion.	Leadership takes CHATO. Team (4). Informed Stakeholders. All staff. CHATO posters. Self-paced. **Onsite discussions.** Lead virtual discussion. Added to orientation materials. Policy changes. Planning booster sessions.	Leadership takes CHATO. Team (5) including **Social worker.** All staff. Self-paced. Lead virtual discussion. Added to orientation materials.	Leadership takes CHATO. Team (4). All staff. CHATO posters. Self-paced. **Onsite discussions.** Lead virtual discussion. **Hand in printed certificate.** Planning booster sessions.	All staff. One module per week. **Onsite discussions.** Lead virtual discussion. **Reward―Food.** **Public recognition.** **Facebook chat.** **Created layton posters in addition to CHATO posters.**

*Notes:* CAN = certified nursing assistant; CHATO = Changing Talk: Online Training; DON = director of nursing; NH0 = feasibility nursing home, NH = Nursing home; RN = registered nurse. Bolded strategies indicate possible facilitators used by the nursing home.

^a^Denotes statistically significant improvement in the CHATS score from pre to posttraining indicating knowledge gain at posttest. See Williams et al. (2021) for more information on CHATS.

^b^Wait-list control NHs.

#### NH leadership evaluation

CHATO evaluation surveys were given to both the NH administrators (*N* = 6) and Implementation Leads (*N* = 6) to compare their perspectives. Overall, the evaluation of CHATO was positive (Tables 8 and 9). Both the Implementation Leads and the administrators agreed NH leadership across the NH were modeling communication learned from the training (mean scores of 70.8 and 72.5 for Implementation Leads and administrators, respectively), and staff were also using the strategies they had learned (mean scores of 65.8 and 70.0). Both the Implementation Leads and the administrators agreed the communication culture across the NH had changed for the better (mean scores of 68.5 and 65.3), and the CHATO training was a good use of their time (mean scores of 73.5 and 74.5). Two thirds of the Implementation Leads and half of the administrators indicated that CHATO was hard to implement. However, two thirds of the Implementation Leads and all the administrators would recommend CHATO to colleagues.

**Table 8.** Implementation Lead Evaluation Survey

Survey question	Mean (*SD*)	NH0	NH1	NH2^a^	NH3	NH4^a^	NH5	NH6^a^	NH7
Role		DON	CNA	^b^	DON	^b^	DON	RN	DON
The CHATO training was hard to implement.	67.3 (22.8)	75	50	^b^	81	^b^	31	73	94
The CHATO training was a good use of our time.	73.5 (24.3)	50	86	^b^	40	^b^	100	94	71
NH staff are using communication strategies they learned.	65.8 (9.7)	59	50	^b^	67	^b^	76	73	70
The NH leadership model the communication strategies.	70.8 (18.9)	73	50	^b^	79	^b^	100	73	50
Communication between staff/residents has improved.	66.5 (9.6)	62	50		65		76	72	73
Communication culture changed for the better across the NH.	68.5 (10.6)	67	50	^b^	65	^b^	78	73	72
Why NH participated in CHATO training?				^b^		^b^			
We want to improve our communication with residents.	*n* = 2			^b^	X	^b^	X		
We want to provide more person-centered care to our residents.	*n* = 2		X	^b^		^b^			X
We need new approaches to address BPSD.	*n* = 1			^b^		^b^		X	
We are working on our Quality Improvement Plan	*n* = 1	X		^b^		^b^			
The CHATO training assisted our NH in reaching this goal.	67.2 (11.3)	66	50	^b^	58	^b^	76	77	76
I plan to recommend this training to colleagues.	Yes (67%)	No	Yes	^b^	No	^b^	Yes	Yes	Yes

*Notes:* BPSD = behavioral and psychological symptoms of dementia; CHATO = Changing Talk: Online Training; CNA = certified nursing assistant; DON = director of nursing; NH = nursing home; RN = registered nurse; *SD* = standard deviation. Implementation evaluation survey included eight items scored on a sliding scale with a range of 1–100 (1–20 = strongly disagree; 21–40 = disagree; 41–60 = neither agree nor disagree; 61–80 = agree; 81–100 = strongly agree).

^a^Wait-list control NHs.

^b^Implementation Lead was the administrator.

**Table 9.** Administration Evaluation Survey

Survey question	Mean (*SD*)	NH0	NH1	NH2^a^	NH3	NH4^a^	NH5	NH6^a^	NH7
Role		Admin	Admin	Admin	^b^	Admin	^b^	Admin	Admin
The CHATO training was hard to implement.	51.0 (13.6)	59	28	50	^b^	50	^b^	69	50
The CHATO training was a good use of our time.	74.5 (21.3)	56	100	50	^b^	70	^b^	100	71
NH staff are using communication strategies they learned.	70.0 (13.0)	65	90	50	^b^	74	^b^	71	70
The NH leadership model the communication strategies.	72.5 (18.4)	76	100	50	^b^	83	^b^	71	55
Communication between staff/residents has improved.	70.8 (16.3)	69	100	50	^b^	66	^b^	72	68
Communication culture changed for the better across the NH.	65.3 (19.9)	72	100	50		70		50	50
Why NH participated in CHATO training?									
We need new approaches to address BPSD.	*n* = 3				^b^	X	^b^	X	X
We want to improve our communication with residents.	*n* = 2	X	X		^b^		^b^		
We are working on our Quality Improvement Plan	*n* = 1			X	^b^		^b^		
The CHATO training assisted our NH in reaching this goal.	70.5 (17.9)	79	100	50	^b^	74	^b^	60	60
I plan to recommend this training to colleagues.	Yes (100%)	Yes	Yes	Yes	^b^	Yes	^b^	Yes	Yes

*Notes:* BPSD = behavioral and psychological symptoms of dementia; CHATO = Changing Talk: Online Training; NH = nursing home. Evaluation survey included eight items scored on a sliding scale with a range of 1–100 (1–20 = strongly disagree; 21–40 = disagree; 41–60 = neither agree nor disagree; 61–80 = agree; 81–100 = strongly agree).

^a^Wait-list control NHs.

^b^Administrator did not participate in implementation.

Leaders were asked about their motivation to participate in the research in the evaluation survey. The Implementation Leads wanted to improve their communication (*n* = 2) with residents and provide more person-centered care (*n* = 2). Additionally, Leads wanted new approaches to address BPSD (*n* = 1) and work on quality improvement (*n* = 1). Sixty-seven percent agreed that CHATO had assisted them in reaching these goals. The administrator’s motivations were slightly different. More of them wanted new approaches to address BPSD (*n* = 3) and improve communication with residents (*n* = 3). One was interested in quality improvement. All the administrators agreed CHATO had assisted them in reaching these goals. Finally, when comparing primary outcome results to evaluations, NHs without significant improvements on the primary outcomes, the CHATS and communication rating, rated CHATO lower when evaluating the program. They also had fewer staff working on implementation.

The leadership interviews were not completed due to the COVID-19 pandemic. They were attempted in April 2020, but Administrators did not have time to complete the phone interviews due to the crisis. However, open-ended survey questions from the evaluation survey identifying barriers and facilitators provided some insight. Barriers they experienced included technical issues with the training platform and difficulty navigating through the modules. Others felt the knowledge test was too difficult and three modules were too time consuming. Another observation was how difficult it is to reliably communicate with staff and how this negatively impacts completion rates. The best strategies identified were rewards, advertising the training through posters and word of mouth, and using the completion certificate as a tracking and evaluation tool.

## Discussion

This study had a 2-phased approach: (a) developing an accessible, remote implementation, and process evaluation for CHATO while engaging NH leadership; ensuring consistent and adaptable application across NHs; and capturing organizational factors and the strategies chosen by each NH; and (b) pilot testing the implementation and process evaluation to prepare for a national pragmatic clinical trial.

We found remote implementation for CHATO was feasible and successful. Leadership engagement was needed to drive successful implementation, and an identified team with champions was better than access to online training alone. Programs with both top–down and bottom–up leadership and organization-wide reinforcement were better than self-motivated training alone. NH leadership utilized the toolkit and other materials to successfully implement CHATO, and those who were more invested in implementing the training saw higher staff engagement and better outcomes overall. Technical assistance was vital as it provided weekly participation and completion rates to keep leadership informed and staff on track. Data collection methods, fidelity tracking, and evaluation were successful and easily scalable to a larger trial.

Additional facilitators included the planning grant allowing us time and structure to create materials and gain insight from NH staff and consultants in the field. Developing a range of implementation materials allowed consistent application with flexibility; providing a mechanism to test the most effective strategies across NHs as they related to the primary outcomes in the national trial. The website was an effective way to have NHs download materials and provided visibility and legitimacy. The communication plan, which included examples for reminding staff and printable posters, was an easy and low-cost way to advertise the training and engage staff. The implementation toolkit provided structure and guidance for NHs while allowing for individual tailoring and innovation. We also found being more prescriptive about which implementation strategies might work was also a motivator and facilitator.

Barriers we encountered at the staff level included computer literacy or access and staff time for training. NHs have already established online learning systems they use regularly to train staff. Introducing a new learning system was difficult for staff, and frustration with technology seemed to impact evaluation even if the content is viewed positively. Initially, we attempted to provide staff with the link to the training website via email; however, most NH staff did not have work or personal email addresses. We chose instead to have leadership create desktop icons directly linked to the training website, allowing busy staff to click the icon and easily access the training. Additionally, online staff training needs to be accessible on a computer at work with time to complete the training, or alternatively, ensure the training is mobile phone ready as many staff only have mobile phones at home.

Despite a small, nonrepresentative sample of NHs in our pilot, differences in NH characteristics and motivation to participate impacted training participation and completion at the organizational level. For example, even if the NH’s parent company expresses considerable interest, individual NHs within the system varied by motivation and buy in. We also found engaging an implementation team rather than a single leader was more effective. We found that the time staff spend planning or implementing CHATO may also influence outcomes in addition to the type of implementation strategies chosen; and weekly completion rates by staff name need to be provided to leadership to ensure higher participation and completion rates.

When comparing our findings to other intervention studies in NHs, we saw many of the same facilitators identified. These included ease of application into practice with on-the-job reinforcement, champions, strong leadership, and communication and coordination with multiple disciplines (i.e., social worker involvement; Groot Kormelinck et al., 2020; Kuske et al., 2007). Similar barriers identified in other studies were unstable organizations, renovations, high staff turnover, and competing demands on time; however, some of our barriers were unique to online education and remote implementation (Groot Kormelinck et al., 2020; Kuske et al., 2007). Ultimately, organizational readiness, leadership engagement, and ongoing training with practice were identified as the keys to successfully modifying staff behaviors (Pimentel et al., 2020). To improve CHATO implementation, we plan to incorporate additional ideas from Gitlin et al. (2020) including the Implementation Climate Scale, a readiness assessment, to measure implementation resources and attitudes toward innovation at the organizational level (Ehrhart et al., 2014; Gitlin et al., 2020). We also plan to expand sustainability by including a 1-year follow up NH survey to capture how NHs adopted practices and maintained changes over time. This will allow development of additional resources to further embed CHATO practices into workflow (Gitlin et al., 2020). The leadership phone interviews and a cost analysis will be completed in the national pragmatic clinical trial.

The ERIC implementation strategy compilation was a useful tool for identifying effective implementation strategies and was easily translated into CHATO strategies for the long-term care setting. Effective ERIC strategies at both the research-level and NH-level were identified in this pilot. At the research-level, Developing a Formal Implementation Blueprint (implementation timeline) and Develop Educational Materials (toolkit and supports) provided organization and support for each NH as they implemented CHATO. Capture and Share Local Knowledge was used to acknowledge the administrator’s and implementation lead’s expertise to gain buy-in and utilize leadership’s knowledge to connect to staff. Make Training Dynamic, Tailor Strategies, and Promote Adaptability were necessary to engage both leadership and staff in participation and completion. These strategies were used within the training itself through interactive activities and behavior-based videos, as well as in the NH environment, by creating a varied, self-selected array of implementation options (self-paced vs. leadership led, implementation team member choice, stakeholder involvement, discussion frequency and type, advertising, recognition, rewards, organizational changes, etc.).

For the development and pilot testing phases, we were interested in identifying which NH-level strategies facilitated implementation processes and had the potential to impact outcomes. Effective implementation strategies for this pilot were identified by examining NHs with significant changes in the primary training outcomes and isolating the unique strategies used. These strategies were linked to ERIC strategies and included: Identify and Prepare Champions (recruiting direct-care champions and taking training first), Create New Clinical Teams (creating diverse implementation teams), Remind Clinicians (reminding weekly with signs, text, email, and/or verbal, and creating custom posters), Intervene To Enhance Uptake and Adherence (onsite discussions or using Facebook chat), and Alter Incentive/Allowance Structures (public recognition, reward, and completion certificate). In the larger trial, implementation analysis will determine the impact of these and other specific strategies on training and resident outcomes to create an evidence-based implementation protocol for CHATO.

## Conclusions

NH staff participation in the pilot varied widely by NH and depended greatly on leadership investment to implement CHATO. Once enrolled, over 63% of the participants completed the training with 87% passing the course. The implementation strategies varied across NHs, and those associated with significant improvements in knowledge gains were assigning champions, including the social worker on the implementation team, utilizing all four mediums (signs, text, email, and verbal) for weekly reminders, giving rewards or public recognition, supporting onsite discussions, and tailoring strategies to their specific NH. Leadership that invested in the training implementation, engaged multiple team members, and varied their implementation strategies had better outcomes overall. Remote implementation will be used to scale up the intervention and test the impact of implementation on CHATO’s primary outcomes in a national pragmatic clinical trial.

## Supplementary Material

igac026_suppl_Supplementary_Material
