# Supplementary material for: Developing and Testing Remote Implementation for the Changing Talk Online (CHATO) Communication Intervention for Nursing Home Staff: A Pilot Pragmatic Randomized Controlled Trial
Source: Innov Aging. 2022 May 2;6(6):igac026. doi: 10.1093/geroni/igac026 (PMC9495503; doi:10.1093/geroni/igac026)
Supplement: igac026_suppl_Supplementary_Material [file igac026_suppl_supplementary_material.docx]

*Research Fidelity Checklist (completed by research team).*

| **CHATO Implementation Strategies** | |
| --- | --- |
| **Planning Phase** | |
| - **Schedule introductory meeting of the CHATO Research Team with Nursing Home Leadership.** | |
|  | - Identify Implementation Team and Champions. - Provide overview of CHATO for Team and Champions (Research team can present or provide educational materials. See Additional Resources on website). - Implementation Team and Champions takes CHATO Training. - Inform stakeholder groups (resident, staff, and family councils; advisory board; industry and professional groups; newsletter and web page, etc.). See suggested materials in toolkit. |
| - **Plan details for all staff to complete CHATO training (three online modules over three weeks) including time, computers, staffing, etc.** | |
|  | - Plan when and where to use staff discussions to reinforce learning: CHATO Virtual Discussion Board (led by Research Team and/or your supervisory staff and champions). Suggestions include:   - One-on-One Staff Discussion with Champion or Supervisor.   - Brief 10-15 minute discussion during staff meetings.   - Group Staff Discussion or Learning Circles - specifically scheduled to discuss CHATO content (See Training Overview under Additional Resources for discussion questions). - Plan rewards and recognition to acknowledge training completion. - Hang posters in hallways or staff areas to advertise and remind staff to take the training. - Plan how staff will access the training via computer, preferably during work hours. |
| - **Provide CHATO link to staff.** | |
|  | - - Plan to remind staff weekly to take the training via email, text, discussion, etc.   - Look for participation and completion rates from the Research Team and follow up with staff. |
| **Training Phase** | |
| - **Remind staff to complete one module each week (See Communication Plan in toolkit).** | |
|  | - - Supervisors/Champions model skills and coach staff in CHATO communication skills.   - Supervisors/Champions ask staff how they are incorporating CHATO skills in their work with residents.   - Target staff members who use elderspeak or other types of ineffective communication and work with them directly to change their communication behavior.   - Complete staff onsite discussion(s).   - Acknowledge/Reward staff completing modules and using CHATO skills. |
| **Follow-up Phase** | |
| - **Recognize and reward staff who completed all three CHATO training modules.** | |
|  | - Add CHATO to onboarding/orientation materials and policy. - Publicize completion of the training to stakeholders (resident, staff, and family councils; advisory board; industry and professional groups; newsletter and web page, etc.). See suggested materials in toolkit. - Supervisors and Champions assess changes in communication and areas for continuing improvement. |
| - **Closing Nursing Home Leadership meeting with Research Team: Complete Surveys and Interview.** | |
|  | - Add CHATO to onboarding/orientation materials and policy - Plan to provide a booster session in 3-6 months using materials provided by Research Team. - Publicize/announce completion of the training to stakeholders (resident, staff, and family councils; advisory board; industry and professional groups; newsletter and websites, etc.). See suggested materials in toolkit. |

*Note.* Key steps for implementing CHATO are marked with bolded text. Suggested strategies for implementation are listed below.
